# An Analysis and Comparison of Survival and Functional Outcomes in Oropharyngeal Squamous Cell Carcinoma Patients Treated with Concurrent Chemoradiation Therapy within City of Hope Cancer Center Sites

**DOI:** 10.3390/jcm9103083

**Published:** 2020-09-24

**Authors:** Rebecca Pharaon, Samuel Chung, Arya Amini, Ellie Maghami, Arnab Chowdhury, Nayana Vora, Sue Chang, Robert Kang, Thomas Gernon, Kelly Hansen, Christina Kelly, Denise Ackerman, Lalit Vora, Sagus Sampath, Erminia Massarelli

**Affiliations:** 1 Department of Medical Oncology and Therapeutics Research, City of Hope National Medical Center, Duarte, CA 91010, USA; rpharaon@coh.org (R.P.); schung@coh.org (S.C.); 2 Department of Radiation Oncology, City of Hope National Medical Center, Duarte, CA 91010, USA; aamini@coh.org (A.A.); NVora@coh.org (N.V.); ssampath@coh.org (S.S.); 3 Department of Head and Neck Surgery, City of Hope National Medical Center, Duarte, CA 91010, USA; emaghami@coh.org (E.M.); rkang@coh.org (R.K.); tgernon@coh.org (T.G.); 4 Department of Computational and Quantitative Medicine, City of Hope National Medical Center, Duarte, CA 91010, USA; achowdhury@coh.org; 5 Department of Pathology, City of Hope National Medical Center, Duarte, CA 91010, USA; suchang@coh.org; 6 Department of Speech and Language Pathology, City of Hope National Medical Center, Duarte, CA 91010, USA; kehansen@coh.org (K.H.); chrikelly@coh.org (C.K.); 7 Department of Clinical Nutrition, City of Hope National Medical Center, Duarte, CA 91010, USA; dackerma@coh.org; 8 Department of Diagnostic Radiology, City of Hope National Medical Center, Duarte, CA 91010, USA; LVora@coh.org

**Keywords:** oropharyngeal cancer, concurrent chemoradiation therapy, human papillomavirus, feeding tube dependency

## Abstract

Oropharyngeal squamous cell carcinoma (OPSCC) is a subset of head and neck cancers that can arise due to human papillomavirus (HPV) infection. We designed a retrospective analysis to determine differences in outcomes of OPSCC patients treated at City of Hope (COH) Cancer Center’s main campus versus selected satellite sites with COH-associated faculty and facilities. Patients diagnosed with OPSCC and treated with concurrent chemoradiation therapy (*n* = 94) were identified and included in the study. Patients underwent treatment at the COH main campus site (*n* = 50) or satellite sites (*n* = 44). The majority of patients were Caucasian, male, and diagnosed with p16 positive stage IV locally advanced OPSCC by AJCC 7th edition. Most patients completed their prescribed cumulative radiation therapy dose and had a complete response to treatment. No significant difference in overall survival and progression-free survival was observed between the main campus and the satellite sites. Our study demonstrates successful treatment completion rates as well as comparable recurrence rates between the main campus and COH-associated satellite sites. A trend toward significant difference in feeding tube dependency at 6-months was observed. Differences in feeding tube placement and dependency rates could be addressed by the establishment of on-site supportive services in satellite sites.

## 1. Introduction

Head and neck cancers, a heterogenous group of cancers originating in the head and neck region, are strongly linked to risk factors such as tobacco use, alcohol consumption, and human papillomavirus (HPV) infection. Oropharyngeal squamous cell carcinomas (OPSCC) are one of the most common head and neck cancers; however, HPV-associated OPSCCs denote a distinct subtype of oropharyngeal cancer with rising incidence compared to the smoking-associated counterpart [1,2]. It has been shown that HPV-associated OPSCC has better prognosis [3,4,5,6,7] and is less likely to have a second primary site [8], but the occurrence of distant metastases is not significantly different than HPV-negative cancer [7,9].

Due to the association between OPSCC and HPV-mediated oncogenesis, the 8th edition of the American Joint Committee on Cancer (AJCC) denoted specific staging criteria and standard treatment for HPV-associated OPSCC, as well as emphasized the importance of extranodal extension (ENE) when determining staging and treatment for patients [10]. Standard treatment generally includes a multimodality approach consisting of transoral robotic surgery (TORS) for early stages or resectable tumors, and radiation therapy with or without concurrent chemotherapy as adjuvant or definitive treatment depending on tumor (T) and nodal (N) disease or high-risk pathologic features such as ENE or carcinoma-involved margins. In the incurable recurrent or metastatic (R/M) OPSCC setting, systemic treatment options include immune checkpoint inhibitors plus or minus chemotherapy with platinum/5-fluorouracil depending on PD-L1 expression, and novel approaches in immunotherapy combinations and HPV vaccines are currently undergoing investigation [11,12].

Cancer patient outcomes after treatment at academic versus satellite sites have been previously explored in esophageal cancer, soft tissue sarcoma, and laryngeal cancer, reporting a trend of improved survival for patients treated at high-volume teaching facilities [13,14,15]. In choosing their treatment center, patients have to account for the geographical location, financial cost, and insurance-approved providers, which can greatly limit their options. Previous studies have examined head and neck cancer outcomes in patients treated at academic centers versus community sites with controversial results [16,17,18]. Currently, academic center and community clinic affiliations have increased to allow patients to have access to high quality and standardized care. However, outcomes analysis has not been studied in partnered academic and satellite sites operating under the same umbrella.

At our institution, City of Hope (COH) Comprehensive Cancer Center, we have established various satellite sites distributed throughout the Southern California region in order to accommodate geographic restrictions and traffic limitations for our patients. Due to the complex nature of treating head and neck cancers, the coordination of various treatments and providers requires precise and effective medical practice. Functional and survival outcomes are dependent on the timing of adjuvant or definitive treatment as well as dedicated follow-up of patients with adequate supportive service such as speech pathology, nutrition specialists, and physical and occupational therapy. Several quality assurance measures are in place at COH, including reviews of satellite site radiation treatment plans performed at the main campus prior to treatment start.

We designed a retrospective study to analyze and compare survival and functional outcomes in patients treated at COH’s main campus versus selected satellite sites with COH-associated faculty and radiation therapy facilities.

## 2. Materials and Methods

### 2.1. Patient and Study Criteria

A retrospective analysis of patients diagnosed with OPSCC and treated with concurrent chemoradiation therapy (CRT) was conducted at COH main campus and selected satellite sites. Main eligibility criteria included a diagnosis of OPSCC, age 18 years or older, definitive/adjuvant treatment including concurrent CRT at a COH site between February 2009 to February 2017, available demographic, treatment, and survival data, functional outcomes, and recent follow-up at a COH site within a year. We utilized our institutional electronic medical record (EMR) to analyze 400 consecutive head and neck cancer patients from various COH sites to enroll in our study. Of the initial 400 patients reviewed, only 94 met the inclusion criteria including OPSCC diagnosis, recent follow-up, and history of CRT treatment at a COH site. Confounding factors considered were age, gender, and T stage. The study was conducted in accordance with the Declaration of Helsinki, and the protocol was approved by the COH institutional review board (#19010).

The primary endpoint was overall survival (OS) of patients treated at the main COH campus versus patients treated at selected COH satellite sites, including South Pasadena, Rancho Cucamonga, Antelope Valley, and Santa Clarita. Secondary endpoints included treatment responses, feeding tube dependency, weight loss, and narcotic use.

### 2.2. Statistical Considerations

The primary analysis of this retrospective study design is to determine the differences in outcomes of OPSCC patients treated at COH Cancer Center main campus versus selected COH-associated satellite sites. Before carrying out inferential statistical procedures, exploratory data analysis was performed. Patient characteristics, including age, gender, race, smoking status, stage at diagnosis, cancer site, and p16 status by immunohistochemistry (IHC), and treatment variables, including chemotherapy agent selected, and dose reduction or interruption, and treatment responses were summarized using descriptive statistics. For continuous variables, means/medians, standard deviations, range and for categorical variables, patient counts, and percentages were provided by cancer center sites (Table 1 and Table 2). Two sample mean/proportion tests (one-sided/two-sided) were performed for quantitative variables. To test homogeneity (equivalent to testing independence) between the main campus and the COH-associated satellite cancer center sites grouped by categorical patient characteristics and treatment outcomes, Pearson’s chi-square test was performed. Fisher’s exact test was performed as an alternative where the expected cell counts were small (less than 5). PEG Tube replacement and feeding tube dependency were also compared between two groups (sites).

Survival estimates were calculated based on the Kaplan-Meier product-limit method, and 95% confidence intervals were calculated using the “exponential” Greenwood variance (log-log transformation) estimate. Differences between Kaplan-Meier curves were assessed by the log-rank test. OS was measured from the date of diagnosis to death from any cause. Patients who were alive at the last contact date were censored. Progression-free survival (PFS) was measured from the date of diagnosis to disease progression or death, whichever occurred first. All calculations were performed using RStudio 1.2.5033; study data were locked for analysis on 15 January 2020.

## 3. Results

### 3.1. Patient Characteristics

**Figure 1:**
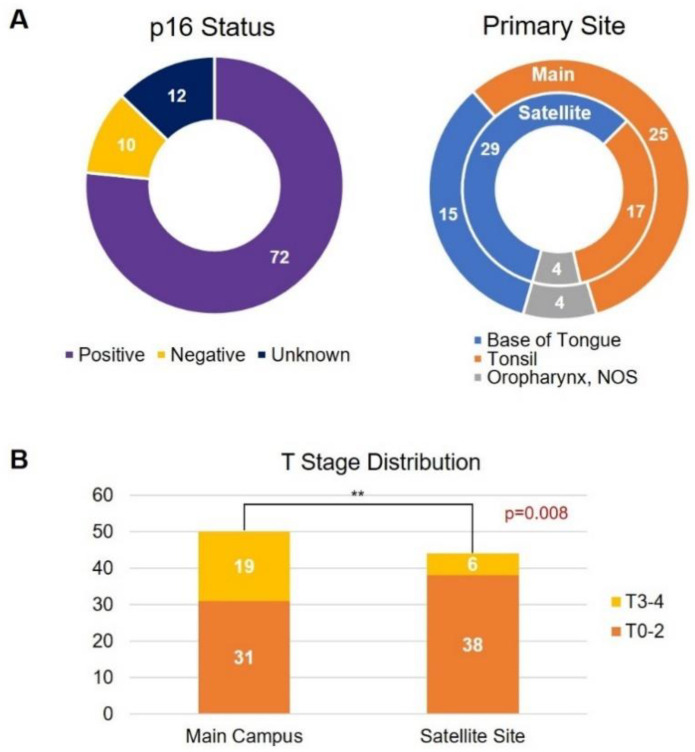
(**A**) P16 breakdown and oropharynx primary site breakdown in patient population and (**B**) T stage disease distribution by treatment site.

94 patients met the inclusion criteria and were included in this retrospective analysis of patients treated between 2009 and 2017 (50 patients at the main COH site and 44 at the satellite sites). Clinicopathologic patient characteristics are presented in Table 1. Majority of patients were p16 positive (*n* = 72, 77%) with primary sites including base of tongue, tonsil, or oropharynx not otherwise specified (Figure 1A). The variables were well-balanced between the two groups, except for T stage demonstrating a significantly higher rate of T3/4 disease treated at the main campus and T0/T2 disease treated at the satellite sites (*p* = 0.008, Table 1 and Figure 1B).

### 3.2. Treatment

All patients were treated with CRT either as definitive or adjuvant therapy. Concurrent chemotherapy agents included cisplatin 40 milligrams per meter squared (mg/m^2^) weekly, high dose cisplatin 100 mg/m^2^ every 3 weeks, carboplatin AUC 2 weekly, cetuximab 400 mg/m^2^ loading dose followed by 250 mg/m^2^ weekly, and platinum/paclitaxel (carboplatin AUC 2 with paclitaxel 50 mg/m^2^ weekly or cisplatin 20 mg/m^2^ with paclitaxel 30 mg/m^2^) (Table 2). A small subset of patients (5 [10%] at the main campus vs. 2 [5%] at the satellite sites) were initially treated with induction chemotherapy (TPF—docetaxel, cisplatin, and fluorouracil) followed by CRT with cisplatin, carboplatin, or cetuximab. Cisplatin was highly favored at the main campus (*n* = 39, 78%) with the rest receiving cetuximab (*n* = 6, 12%), carboplatin (*n* = 4, 8%), and carboplatin/paclitaxel (*n* = 1, 2%). In contrast, cisplatin and cetuximab use was equally distributed at the satellite sites (*n* = 21, 48% each), while the remaining two patients either received carboplatin or cisplatin/paclitaxel. Of the 60 cisplatin-treated patients, 58 (98%) received the recommended total minimum dose of 200 mg/m^2^ with two patients (2%) receiving less due to prior surgical resection/physician’s choice.

All patients were treated with intensity modulated radiotherapy (IMRT) and the median radiation therapy dose was 68.8 Gray (Gy) (50 to 70 Gy) given at 2 Gy per fraction. A subset of patients at both sites (*n* = 23, 25%) underwent surgical resection with TORS prior to concurrent CRT. This subset of patients was subsequently treated with adjuvant CRT and given an average radiation dose of 65.3 Gy (50 to 66 Gy), as determined by pathologic risk features and performance status. Average length of radiotherapy treatment was 48 days at the main campus and 50 days at the satellite sites. Forty-nine (98%) patients at the main campus and 43 (98%) patients at the satellite sites completed their prescribed radiation therapy dose. Patients experienced common CRT effects as reported by the treating physician including mucositis, skin toxicities, hearing loss, neuropathy, nausea/vomiting, consistent with typical acute toxicities from CRT; however, proper grading of toxicity was not available except for weight loss (Table 2).

Five patients at the main campus switched chemotherapy drugs from high-dose cisplatin to carboplatin or cetuximab, and two patients at the satellite sites switched from high dose cisplatin to carboplatin and from cetuximab to weekly cisplatin due to adverse symptoms secondary to the original chemotherapy. The reasons for chemotherapy drug switch included tinnitus, elevated creatinine, and azotemia. Overall, the chemotherapy dose was reduced by at least 20% in seven patients (four at the main campus vs. three at the satellite sites, *p* = 0.83) treated with high-dose cisplatin (100 mg/m^2^) due to adverse symptoms. The number of patients who missed a therapy dose(s) or had chemotherapy treatment interruptions because of significant treatment-related adverse events (i.e., azotemia, leukopenia, neutropenia, thrombocytopenia), unplanned hospitalizations, or patient decisions were similar among the COH main campus and satellite groups (11 vs. nine patients, *p* = 0.86). During the course of radiation therapy treatment, seven patients in the main campus and six patients in the satellite sites had treatment interruptions/breaks of at least two days. The reasons for treatment interruptions were related to severe weight loss (*n* = 2), thus requiring another radiotherapy treatment planning computed tomography scan, toxicities secondary to treatment (*n* = 7), insurance issues (*n* = 1), patient’s choice (*n* = 1), and unplanned hospitalizations (*n* = 2). Reasons for unexpected hospitalization (six at the main campus vs. four at the satellite sites) were related to significant treatment-related adverse events such as severe dehydration, severe adverse infusion reaction to cetuximab, aspiration pneumonia, G-tube cellulitis, or pulmonary embolism.

The percentage of patients who completed the prescribed full treatment dose of both chemotherapy and radiation therapy without interruptions of radiation therapy was 86% (*n* = 43) at the main campus and 82% (*n* = 36) at the satellite locations.

### 3.3. Survival Outcomes

Patients underwent restaging imaging 12 weeks after completion of treatment to measure their response to treatment. Thirty-five (70%) patients achieved a complete response (CR) to treatment, 13 (26%) patients had a partial response (PR), and two (4%) patients had progression of disease (POD)/persistent disease at the main center site. Thirty-seven (84%) patients achieved a CR, five (11%) patients had a PR, and two (5%) patients had POD/persistent disease at the satellite sites. The patients with POD/persistent disease were subsequently treated with pembrolizumab at the main cancer site, or further lines of chemotherapy (carboplatin/paclitaxel, carboplatin/gemcitabine, cetuximab) followed by nivolumab or pembrolizumab at the satellite sites.

**Figure 2:**
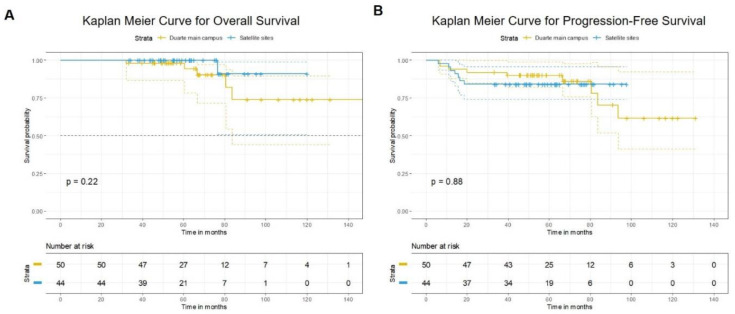
(**A**) Kaplan-Meier curve for overall survival by treatment site and (**B**) Kaplan-Meier curve for progression-free survival by treatment site.

With a follow-up of 140 months (~12 years), median OS was not yet reached for both the main campus and the satellite sites (Figure 2A). Between both sites, there was no significant difference in OS (*p* = 0.22) and PFS (*p* = 0.88) (Figure 2A,B). No significant difference in two-year (47 [94%] vs. 37 [84%] patients, *p* = 0.12) and five-year (46 [92%] vs. 37 [84%] patients, *p* = 0.23) PFS was observed between the main campus and the satellite sites, respectively (Table 2).

### 3.4. Functional Outcomes

Modified Barium Swallow Studies (MBSS) were performed more frequently at the main cancer center than the satellite sites due to the presence of a comprehensive team of speech pathologists. 12 patients (24%) underwent feeding tube placement at the main campus, prophylactically (*n* = 4) or during treatment (*n* = 8). Two of the 12 patients continued to be feeding tube dependent after 6 months. Twenty-four patients (55%) underwent feeding tube placement at the satellite sites, prophylactically (*n* = 20) or during treatment (*n* = 4). Eleven of the 24 patients continued to be feeding tube-dependent at 6 months. Prophylactic feeding tube placement was associated with higher rates of 6-month feeding tube dependency (*n* = 2, 100% at main site, *n* = 9, 92% at satellite sites). Weight loss was recorded throughout CRT treatment and during the 6-month post-treatment follow-up. Thirty-seven patients (74%) lost ≤10% baseline weight during treatment while the remaining 13 (26%) lost >10% of their initial body weight at the main site. Twenty-six (59%) patients lost ≤10% baseline weight, while the remaining 18 (41%) lost >10% of their baseline weight during treatment at the satellite sites. Twenty-four patients (48%) lost ≤10% baseline weight at the 6-month post-treatment follow-up, while 26 (53%) lost >10% of their initial body weight at the main site. Fourteen patients (32%) and 30 patients (68%), respectively, lost ≤ and >10% of their initial body weight at the 6-month post-treatment follow-up at the satellite sites. Overall, at 6 months after completion of treatment, both sites noted an increased number of patients experiencing >10% baseline weight loss (13 to 26 patients at the main campus and 18 to 30 patients at the satellite sites). Of the 12 patients who underwent feeding tube placement at the main site, 4 (33%) and 7 (58%) patients lost >10% baseline weight during treatment and at 6 months post-treatment, respectively. Of the 24 patients who underwent feeding tube placement at the satellite sites, eight (33%) and 16 (66%) patients lost >10% baseline weight during treatment and at 6 months post-treatment, respectively.

Narcotic use was recorded at 3- and 6-month timepoints after completion of radiation therapy. At the main center, 16 (32%) patients had recorded persistent use of narcotics at a 3 months post-treatment interval and 9 (18%) patients required persistent narcotic use at the 6-month mark. At the satellite sites, a similar number of patients (*n* = 13, 30%) reported narcotic use 3 months post-treatment, and 10 (23%) patients registered persistent narcotic use at the 6-month mark.

## 4. Discussion

We conducted a retrospective analysis of HPV-positive OPSCC patients treated with definitive or postoperative CRT at a large NCI-designated comprehensive cancer center (COH) main campus and compared it to selected institutional satellite sites with availability of dedicated radiation therapy facilities staffed with COH faculty with primary endpoint of OS and secondary endpoints including treatment response, feeding tube dependency, and weight loss throughout treatment and 6 months post-treatment. Our analysis is the first to compare patient outcomes in a comprehensive NCI designated cancer center and its affiliated satellite sites. COH main site and satellite sites demonstrated OS rates consistent with already published favorable survival rates in OPSCC treated with multimodality therapy approach [7,19], while median OS at ~12 years was not reached (Figure 2A). No significant difference in two-year (94% vs. 84%, *p* = 0.12) and five-year (92% vs. 84%, *p* = 0.23) PFS was observed between the main campus and the satellite sites (Table 2). Our data did not align with previously reported analyses of head and neck cancer outcomes in academic centers versus community sites that demonstrated superior survival rates in patients treated at academic institutions [16,18]. One major difference between our study and the previously mentioned analyses [16,18] relates to the fact that our patients were treated under an umbrella institution, COH, regardless of treatment center location. Overall, our institution takes strides to standardize patient care across all sites by the longstanding establishment of weekly radiation oncology web rounds dedicated to reviewing all radiation treatment plans at any COH site. Our outcomes are possible because of these efforts of treatment compliance between main and satellite centers where there is some uniformity in dose delivery, nodal coverage based on primary site and nodal disease distribution, overall treatment volumes, and established guidelines for treatment planning. Furthermore, the medical oncologists at the COH satellites are part of the medical oncology main department and are required to attend quarterly retreats with the oncologists at main campus.

Regarding differences in the choice of concurrent systemic therapy, the oncologists at the main campus site strongly favored cisplatin as the chemotherapy agent of choice (78%), whereas satellite site medical oncologists equally favored cisplatin (48%) and cetuximab (48%) when treating their patients (*p* = 0.001). Considering the different distribution of tumor size between the two groups of patients with main campus seeing a higher number of T3–4 disease (19 [38%] vs. six [14%], *p* = 0.008) (Table 1 and Figure 1B), the choice of cisplatin-based therapy could have been influenced by the more advanced stage of disease at presentation. In addition, these patients were treated before the completion and publication of The Radiation Therapy Oncology Group (RTOG) 1016 clinical trial that compared systemic therapy agent cisplatin versus cetuximab, a monoclonal antibody against EGFR, in HPV-positive OPSCC, and reported superior PFS with cisplatin over cetuximab (78% vs. 67%, *p* < 0.001) [20]. A small subset of patients underwent treatment with induction chemotherapy followed by CRT in our analysis (five at the main campus vs. two patients at the satellite sites, *p* = 0.31). While the use of induction chemotherapy in head and neck cancers is controversial, a recent phase II trial demonstrated feasibility of different induction regimens stratified by disease risk in locally advanced head and neck squamous cell carcinoma [21].

In our study, chemotherapy dose reductions occurred among patients who were treated with high-dose cisplatin, underscoring the heavy burden of side effects that this treatment carries and the need for continuous monitoring by the medical oncologist to avoid permanent renal and neurological impairment effect on quality of life and survival outcomes. The majority of patients completed their prescribed cumulative radiation therapy dose (*n* = 94, 98%). The rate of treatment interruptions and hospitalizations were comparable between the two groups of patients when adjusted by systemic treatment choice (Table 2). Chronic narcotic use at 3 months did not differ between the two groups (32% at the main campus vs. 30% at the satellite sites, *p* = 0.98) (Table 2), demonstrating a significant number of patients persistently using narcotics after completing treatment. Chronic narcotic use was trending downwards at 6 months, and is in line with previously reported data in patients with OPSCC [22].

**Figure 3:**
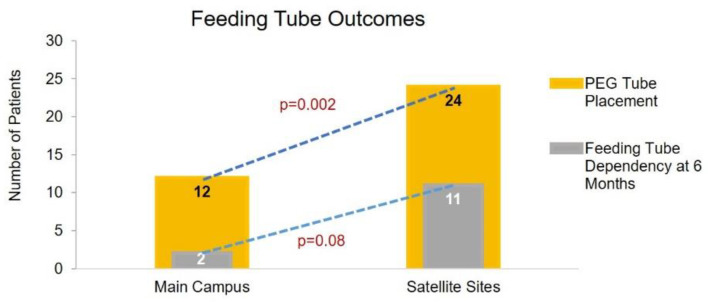
Feeding tube placement and 6-month dependency by treatment site.

Our retrospective analysis demonstrated a significant difference in the rate of feeding tube placement between the main campus and satellite sites (24% vs. 55%, *p* = 0.002). Of those patients, four (33%) at the main campus and 20 (83%) at the satellite sites underwent prophylactic tube placement (*p* = 0.003). A trend towards significance in feeding tube persistence at 6 months was observed (17% vs. 46%, *p* = 0.08) (Table 2 and Figure 3).

MBSS were more likely to be performed at the main campus versus satellite sites given the availability of on-site speech pathology at the main campus. Multiple studies have been conducted to determine the advantage of prophylactic versus reactive PEG tube placement and the results are controversial [23,24,25,26,27,28,29]. However, studies have reported the significance of prophylactic PEG tube placement on enteral feed dependency rates [23,24,29], consistent with our feeding tube outcomes. This difference highlights the need for supportive service availability at satellite sites that could be potentially reached with remote consultation with institutional dedicated nutritionists and speech pathologists at our COH satellite centers. There was no significant difference in the degree of weight loss between the two groups of patients with the majority maintaining a ≤10% baseline weight loss (75% vs. 59%, *p* = 0.12); however, this could be the result of a higher prophylactic feeding tube rate in the satellite centers than main campus (Table 2).

Overall, patients who are at a higher risk of recurrence due to poor pathologic features, comorbidities, and nutritional status should be identified earlier during treatment so that they can have access to supportive services. At our institution, we are working on directing those patients earlier on to speech pathologists and nutritionists to eventually identify ones who might benefit from treatment modifications or early implementation of dietary needs. Furthermore, we are exploring the feasibility of implementing a program that allows for an initial comprehensive speech and nutrition evaluation at the main campus with continued telehealth follow-ups in the satellite sites and return to the main campus for repeat instrumental testing when necessary. This would reduce the need of in-person visits with speech pathologists and nutritionists and we plan to understand if this initiative could be effective in improving the 6 months feeding tube persistence rate. Due to the COVID-19 pandemic, numerous institutions have implemented telehealth services in order to accommodate patients and decrease needless exposure. Our institution offers outpatient telemedicine consults and follow-up visits via telephone or video call, and this service has been extended to our satellite sites. In the future, this implementation will greatly benefit patients undergoing treatment at satellite sites that need speech pathology and nutritionist oversight but do not have access to the main academic center, in order to minimize unnecessary feeding tube placements and ensure good functional outcomes.

Precision medicine in oropharyngeal cancers is difficult to accomplish as we do not select treatment based on molecular targets other than HPV status. Currently, HPV status is the only biological feature on which we establish disease risk and prognosis. We clearly understand that there is a need for molecular-driven treatment selection in the future. Due to EMR integration between the main campus and satellite sites, a wide range of patient information is readily accessible, thus allowing for academic and community physicians to efficiently collaborate on research studies. Based on this joint retrospective analysis, we are conducting and examining the molecular status of these academic and community patients to try to understand if we can translate meaningful results.

We recognize that our study contains limitations including intrinsic bias of patient selection in a retrospective analysis and limitations of our EMR search which identified our cohort of patients. We designated the HPV-associated OPSCC according to p16 IHC status, based on the evidence of concordance between p16 IHC staining and HPV in situ hybridization in HPV-associated oropharyngeal carcinomas that makes p16 IHC a surrogate marker for HPV infection [30]. The discrepancies between our study in OPSCC and those who observed improved survival in head and neck cancer patients treated at an academic institution could be attributed to the excellent prognosis of HPV-positive OPSCC. Disparities in disease stage, socioeconomic factors, patient location, and insurance play a role in the different characteristics of patients treated at the main campus compared to the satellite sites. Worse prognosis primary sites such as oral cavity and larynx could potentially demonstrate differences in survival.

In conclusion, our study highlights the importance of the necessity to treat OPSCC in tertiary cancer centers and their associated satellite network with availability of dedicated and experienced radiation and medical oncologists and up-to-date facilities that allow continuous communication between physicians and supportive services including speech pathology and nutrition. We are currently designing a prospective study to assess the implementation of telemedicine for supportive services in the satellite sites. This is of great importance as poor functional outcomes represent a heavy burden for our current HPV-positive OPSCC population that is young, very functional, and deserves high standards of post-treatment quality of life.

## Figures and Tables

**Table 1:** Patient Characteristics by Treatment Site.

		Main Campus (*n* = 50) *n*, %	Satellite Sites (*n* = 44) *n*, %	*p*-Value
Age median		58	61	
Gender	Female	10 (20)	5 (11)	*p* = 0.25
	Male	40 (80)	39 (89)	
Vital Status	Alive	44 (88)	43 (98)	*p* = 0.12
	Deceased	6 (12)	1 (2)	
Race	Caucasian	38 (76)	32 (73)	*p* = 0.08
	African American	1 (2)	1 (2)	
	Asian	8 (16)	3 (7)	
	Native American	1 (2)	0 (0)	
	Other/Unknown	2 (4)	8 (18)	
Smoking status	Never	25 (50)	22 (50)	*p* = 1.00
	Former/Current	25 (50)	22 (5)	
AJCC 7th Edition Stage	I	0 (0)	0 (0)	*p* = 0.24
	II	2 (4)	2 (5)	
	III	5 (10)	9 (20)	
	IV	43 (86)	33 (75)	
T stage	T0–2	31 (62)	38 (86)	*p* = 0.008
	T3–4	19 (38)	6 (14)	
N stage	N0–2a	14 (28)	19 (43)	*p* = 0.124
	N2b-N3	36 (72)	25 (57)	
M stage	M0	50 (100)	44 (100)	—
	M1	0 (0)	0 (0)	
Cancer site	Tonsil	17 (34)	25 (57)	*p* = 0.06
	Base of Tongue	29 (58)	15 (34)	
	Oropharynx, NOS	4 (8)	4 (9)	
P16 status	Positive	42 (84)	30 (68)	*p* = 0.18
	Negative	4 (8)	6 (14)	
	Unknown	4 (8)	8 (18)	

**Table 2:** Treatment and functional outcomes.

		Main Campus *n*, %	Satellite Sites *n*, %	*p*-Value
Chemotherapy agent	Cisplatin	39 (78)	21 (48)	*p* = 0.001
	Carboplatin	4 (8)	1 (2)	
	Cetuximab	6 (12)	21 (48)	
	Platinum/Paclitaxel	1 (2)	1 (2)	
Chemotherapy drug change		5 (10)	2 (5)	*p* = 0.31
Chemotherapy interruption/missed dose		11 (22)	9 (20)	*p* = 0.86
Chemotherapy dose reduction		4 (8)	3 (7)	*p* = 0.83
Radiation therapy interruption		7 (14)	6 (14)	*p* = 0.96
PEG tube placement	Yes	12 (24)	24 (55)	*p* = 0.002
	No	38 (76)	20 (45)	
Prophylactic PEG tube placement		4 (33)	20 (83)	*p* = 0.003
PEG tube dependency	>6 months	2 (17)	11 (46)	*p* = 0.08
Weight loss during treatment	≤10%	37 (74)	26 (59)	*p* = 0.12
	>10%	13 (26)	18 (41)	
Weight loss baseline to 6 months post-treatment	≤10%	24 (48)	14 (32)	*p* = 0.11
	>10%	26 (52)	30 (68)	
Hospitalizations during treatment		6 (12)	4 (9)	*p* = 0.65
Treatment Response	Complete response	35 (70)	37 (84)	*p* = 0.24
	Partial response	13 (26)	5 (11)	
	Progressive/persistent disease	2 (4)	2 (5)	
Recurrent disease		5 (10)	7 (16)	*p* = 0.39
3-month narcotic use	Yes	16 (32)	13 (30)	*p* = 0.98
	No	34 (68)	25 (57)	
	Unknown	8 (16)	6 (13)	
6-month narcotic use	Yes	9 (18)	10 (23)	*p* = 0.83
	No	33 (66)	28 (64)	
	Unknown	8 (16)	6 (13)	
Progression-free survival	2-year	47 (94)	37 (84)	*p* = 0.12
	5-year	46 (92%)	37 (84)	*p* = 0.23
